# Association between social jetlag and diabetes self-management in adults with type 2 diabetes: a cross-sectional study with serial mediation analysis

**DOI:** 10.3389/fpubh.2026.1979499

**Published:** 2026-10-16

**Authors:** Jinman He, Shuaiyou Wang, Jingyu Zhang, Shuang Li, Xiaoting Huang, Chongmei Huang, Minhui Liu

**Affiliations:** 1 School of Nursing, Ningxia Medical University, Yinchuan, China; 2 School of Public Health, Ningxia Medical University, Yinchuan, China; 3 Xiangya School of Nursing, Central South University, Changsha, China

**Keywords:** depressive symptoms, diabetes self-management, sleep quality, social jetlag, type 2 diabetes

## Abstract

**Background:**

Diabetes self-management requires consistent daily behaviors. Social jetlag, the mismatch between workday and free-day sleep timing, may disrupt daily behavior routines. This study examined the association between social jetlag and self-management in adults with type 2 diabetes (T2D), and whether sleep quality and depressive symptoms statistically accounted for portions of this association.

**Methods:**

This cross-sectional study recruited employed adults aged 18–60 years with T2D from the endocrinology departments of three tertiary public general hospitals in Ningxia Hui Autonomous Region, China, from January to April 2026. Social jetlag was calculated from the micro Munich Chronotype Questionnaire; sleep quality, depressive symptoms, and diabetes self-management were assessed using the Pittsburgh Sleep Quality Index, Patient Health Questionnaire-9, and Summary of Diabetes Self-Care Activities, respectively. Multivariable linear regression and serial mediation analysis with 5,000 bootstrap resamples were performed.

**Results:**

A total of 339 participants were analyzed. Each additional h of social jetlag was associated with higher PSQI score (*B* = 1.352, 95% CI 1.042 to 1.663), higher PHQ-9 score (*B* = 3.772, 95% CI 3.314 to 4.229), and lower SDSCA score (*B* = −6.623, 95% CI −7.566 to −5.679). The direct association remained significant after PSQI and PHQ-9 scores were included (*B* = −3.830, 95% CI −5.048 to −2.611). The specific indirect association through depressive symptoms alone was −2.230 (95% bootstrap CI −3.053 to −1.469), and the serial indirect association through sleep quality and depressive symptoms was −0.314 (95% bootstrap CI −0.534 to −0.150).

**Conclusion:**

Greater social jetlag was associated with poorer diabetes self-management. Significant indirect associations were observed through depressive symptoms alone and through the serial pathway from sleep quality to depressive symptoms. These associations do not establish temporal ordering or causal mediation because the data were cross-sectional.

## 1 Introduction

Diabetes affects approximately 589 million adults worldwide (1). Effective diabetes self-management is central to diabetes care (2–5). It includes dietary regulation, physical activity, medication use, blood glucose monitoring, and foot care. Better self-management is associated with fewer hospital admissions and a lower risk of premature mortality (6, 7). However, many adults do not perform these behaviors consistently (8, 9). Identifying potentially modifiable barriers to diabetes self-management is therefore important. Disruption of the sleep–wake rhythm may represent one such barrier.

Misalignment of the sleep–wake rhythm between workdays and free days is known as social jetlag (10). About 65% of adults with T2D experience social jetlag in excess of 1 h (11). Greater social jetlag is associated with less favorable glycaemic and metabolic outcomes (11, 12), insulin resistance, metabolic-syndrome parameters (13), and obesity (14). This association becomes even more significant when considering that effective diabetes self-management requires health behaviors to be performed consistently across all seven days of the week, without distinction between workdays and free days (2, 3). Yet social jetlag, by its very nature, creates a discrepancy in daily routines between these workdays and free days, potentially undermining patients' ability to maintain the consistency needed for optimal self-management (10). Therefore, this shared temporal framework suggests that social jetlag may be an overlooked modifiable barrier in diabetes education and care and warrants further investigation (15).

Beyond identifying this potential barrier, examining factors associated with social jetlag and diabetes self-management could inform nursing assessment and support. Poor sleep quality (16) and depressive symptoms (17, 18) are common among adults with T2D. Sleep quality (19) and depressive symptoms (20, 21) may each account for part of the association between social jetlag and diabetes self-management. The two factors may also be interrelated: greater social jetlag has been associated with depressive symptoms (22), poor sleep quality is associated with depressive symptoms in adults with T2D (23), and associations between social jetlag and mental health have also been reported among resident physicians (24). However, the individual and serial indirect associations involving sleep quality and depressive symptoms in the relationship between social jetlag and diabetes self-management have not been fully characterized in adults with T2D.

This study examined the association between social jetlag and diabetes self-management and whether sleep quality and depressive symptoms statistically accounted for portions of this association in adults with T2D. We hypothesized that greater social jetlag would be associated with poorer sleep quality, more severe depressive symptoms, and poorer diabetes self-management, and that significant indirect associations would be observed through sleep quality and depressive symptoms.

## 2 Materials and methods

### 2.1 Study design and setting

This cross-sectional study was conducted from January to April 2026 in the endocrinology departments of three tertiary public general hospitals in Ningxia Hui Autonomous Region, China: the General Hospital of Ningxia Medical University, Wuzhong People's Hospital, and the People's Hospital of Ningxia Hui Autonomous Region. The study was reported in accordance with the Strengthening the Reporting of Observational Studies in Epidemiology (STROBE) guideline for cross-sectional studies (25) and the completed STROBE checklist is provided. The study was approved by the Medical Ethics Review Committee of Ningxia Medical University (Ethics Approval No. 2025-113), and written informed consent was obtained from all participants.

### 2.2 Participants

Participants were recruited by convenience sampling from the three endocrinology departments.

We included participants who were: (1) aged 18–60 years; (2) diagnosed with T2D by a licensed physician; (3) able to read Chinese and communicate in Mandarin; and (4) currently employed.

We excluded participants who were: (1) engaged in shift or night work in the past three months; (2) diagnosed with severe cardiac or hepatic disease by a licensed physician; or (3) diagnosed with cognitive impairment by a licensed physician.

### 2.3 Sample size calculation

The required sample size was estimated using G^*^Power version 3.1.9.4 (Heinrich Heine University Düsseldorf, Düsseldorf, Germany). Assuming a fixed-model multiple linear regression analysis with 18 predictors, an effect size of *f* ^2^ = 0.10, an α of 0.05, and 95% statistical power, the calculation yielded a minimum sample size of 311. The sample size was increased to 328 to allow for 5% incomplete or invalid questionnaires.

### 2.4 Data collection

Data were collected on site through an electronic questionnaire hosted on the “Questionnaire Star” online platform. Trained investigators approached potential participants in the endocrinology departments, assessed their eligibility (including current employment), and explained the study purpose and procedures. Investigators also emphasized voluntary participation, data confidentiality, and the right to withdraw before data analysis. Individuals who agreed to participate were invited to complete the questionnaire on site.

All items in the online questionnaire were mandatory, and participants could not submit it until every item had been completed. Before data collection, we established three criteria for excluding returned questionnaires with inconsistent responses. After collection, two researchers independently reviewed the returned questionnaires against these predefined criteria. We excluded questionnaires if: (1) the reported diabetes duration exceeded the participant's current age; (2) napping responses were inconsistent (e.g., “no napping” with a duration greater than 0 min, or a napping category with a duration of 0 min); or (3) social jetlag could not be calculated because the participant reported 0 or 7 usual workdays per week.

### 2.5 Measures

The survey included a self-designed questionnaire, the micro Munich Chronotype Questionnaire (μMCTQ), the Pittsburgh Sleep Quality Index (PSQI), the Patient Health Questionnaire-9 (PHQ-9), and the Summary of Diabetes Self-Care Activities (SDSCA).

A self-designed questionnaire collected demographic, clinical, and lifestyle variables, including age, gender, education level, marital status, monthly per-capita income, body mass index, duration of T2D, diabetes-related complications, smoking, drinking, caffeine intake, napping, HbA1c, fasting plasma glucose (FPG), and 2-h postprandial plasma glucose (2-h PPG). Based on the Chinese Guidelines for the Prevention and Treatment of Diabetes (2024 edition), diabetes-related complications included diabetic retinopathy, diabetic kidney disease, diabetic neuropathy, cardiovascular disease, cerebrovascular disease, peripheral arterial disease, and diabetic foot disease (26). Participants reported the number of previously diagnosed complications as 0, 1, 2, or ≥3. We did not record specific complication types or independently verify the diagnoses. Participants provided the most recent HbA1c, FPG, and 2-h PPG results obtained within the three months prior to questionnaire completion, based on their own medical records or laboratory reports.

The μMCTQ was used to assess sleep timing (27). A workday was any day on which the participant worked, and a free day was any day on which the participant did not work, regardless of the day of the week. Participants reported their usual number of workdays per week and sleep-onset and wake-up times separately for workdays and free days. Sleep duration was calculated as the interval between sleep onset and wake time. Weighted mean weekly sleep duration was calculated as [(workday sleep duration × number of workdays) + (free-day sleep duration × number of free days)]/7, and the sleep-duration difference was calculated as free-day minus workday sleep duration. Sleep midpoint was calculated as sleep onset plus half of sleep duration. Sleep durations and midpoints were rounded to two decimal places before social jetlag was calculated. Social jetlag was defined as the absolute difference between free-day and workday sleep midpoints: SJL = |MSF – MSW|. It was treated as a continuous variable and expressed in hours.

The PSQI was used to assess sleep quality over the past month (28). Total scores range from 0 to 21, with higher scores indicating poorer sleep quality. The PSQI also includes an item on the frequency of sleep-aid medication use, but it did not collect data on medication names or types. Cronbach's α of PSQI was 0.755 in this study.

The PHQ-9 was used to assess depressive symptoms during the previous two weeks (29). The total score ranges from 0 to 27, with higher scores indicating more severe depressive symptoms. Cronbach's α for the PHQ-9 was 0.810 in this study.

We assessed diabetes self-management using the authorized Chinese version of the 11-item Summary of Diabetes Self-Care Activities (SDSCA), originally developed by Toobert et al. (30). Permission to use the Chinese version was obtained from Oregon Research Institute through Mapi Research Trust. The 11 items cover general diet (2), specific diet (2), physical activity (2), blood glucose monitoring (2), foot care (2), and medication adherence (1). Specifically, the items address adherence to a diabetes diet during the previous week and month, consumption of at least five types of fruit and vegetables, consumption of high-fat foods or full-fat dairy products, more than 30 min of physical activity, moderate-intensity physical activity, blood glucose monitoring, blood glucose monitoring as recommended by a physician, foot inspection, inspection of the inside of shoes, and use of prescribed medication or insulin (31). Each item was scored from 0 to 7 days per week. The high-fat food item was reverse-scored, and the 11 item scores were summed to yield a total score ranging from 0 to 77, with higher scores indicating better diabetes self-management. A previous Chinese validation study reported an overall Cronbach's α of 0.62 and a test–retest reliability coefficient of 0.83 (31). Cronbach's α of SDSCA was 0.732 in the study.

### 2.6 Statistical analysis

Data were analyzed using IBM SPSS Statistics version 27.0.1 (IBM Corp., Armonk, NY, USA) and the PROCESS macro version 5.0 (developed by Andrew F. Hayes). Descriptive statistics were used to summarize participant characteristics. Continuous variables were presented as median (Q1, Q3), with quartiles obtained using the default percentile calculation in SPSS FREQUENCIES; categorical variables were summarized as *n* (%). Participants were grouped into three groups based on the duration of social jetlag: < 1 h, 1–2 h, and >2 h. Group differences were examined using the Kruskal–Wallis test for continuous variables and Pearson's chi–square test for categorical variables.

Multivariable linear regression was used to examine the associations between social jetlag and sleep quality, depressive symptoms, and diabetes self-management. Covariates were selected based on previous evidence about social jetlag, metabolic factors (11, 13, 14), sleep quality in adults with T2D (16, 19), diabetes self-management and related health behaviors (2). No formal prespecified causal diagram or conceptual framework was developed to guide covariate selection. The covariates included age, gender, body mass index, diabetes duration, self-reported number of diabetes-related complications, smoking frequency, drinking frequency, caffeine intake frequency, and napping pattern. Smoking, drinking, and caffeine intake were coded as ordinal frequency variables (0 = none, 1 = < 1 time/week, 2 = 1–3 times/week, 3 = 4–6 times/week, and 4 = ≥7 times/week). Napping was treated as nominal, with indicator variables for workdays only, free days only, and both days, using no napping as the reference. Sleep quality and depressive symptoms were not included as conventional covariates because both were hypothesized mediators.

Serial mediation analysis estimated direct and indirect associations of social jetlag with diabetes self-management through sleep quality and depressive symptoms. Indirect effects were estimated using PROCESS Model 6 with 5,000 bootstrap resamples and 95% percentile bootstrap confidence intervals, with the random seed fixed at 12,345 (32). For reproducibility, cases were sorted by participant ID before resampling and the SPSS MC random-number generator was used. Reporting followed recommendations for observational mediation research (33, 34).

Multicollinearity was assessed using variance inflation factors (all < 5), and unstandardised coefficients are reported. Eight sensitivity analyses were performed: (1) additional adjustment for education, marital status, and monthly per-capita income, (2) further adjustment for HbA1c, FPG, and 2-h PPG in addition to the sociodemographic variables in analysis (1), (3) additional adjustment for weighted mean weekly sleep duration and the free-day–workday sleep-duration difference, (4) use of a PSQI score excluding the sleep-duration component, (5) use of a PSQI score excluding the sleep-medication component, (6) reversal of the mediator order, with depressive symptoms preceding sleep quality in the statistical model, (7) use of a PHQ-9 score excluding the sleep item, and (8) additional adjustment for recruitment site. Glycaemic indicators were included as a supplementary robustness adjustment rather than established confounders because they may partly lie downstream of sleep timing and self-management. In analysis (8), recruitment site was entered using indicator variables, with the General Hospital of Ningxia Medical University as the reference category, all covariates were retained. A two-sided *P* < 0.05 was considered statistically significant.

## 3 Results

### 3.1 Participant characteristics

A total of 361 questionnaires were returned. After 22 questionnaires were excluded according to the predefined criteria, 339 questionnaires were retained for analysis. Participant characteristics are summarized in Table 1 and Supplementary Table S1. 37.5% (127), 32.7% (111), and 29.8% (101) of participants were recruited from the General Hospital of Ningxia Medical University, Wuzhong People's Hospital, and the People's Hospital of Ningxia Hui Autonomous Region, respectively. Overall, 189 participants (55.8%) were male. Median age was 39.0 (33.0, 44.0) years, and median diabetes duration was 4.0 (2.0, 7.0) years.

**Table 1:** Participant characteristics according to social jetlag group.

Characteristic	Overall (*N* = 339)	< 1 h (*n* = 78)	1–2 h (*n* = 136)	>2 h (*n* = 125)	*P* value
Age, years	39.0 (33.0, 44.0)	42.5 (38.0, 48.3)	39.0 (33.0, 45.0)	36.0 (31.0, 42.0)	< 0.001
Body mass index, kg/m^2^	24.09 (21.77, 26.17)	24.51 (22.46, 26.52)	24.02 (21.49, 26.30)	23.80 (21.20, 25.93)	0.203
Duration of diabetes, years	4.0 (2.0, 7.0)	4.0 (2.0, 6.3)	4.0 (3.0, 7.0)	4.0 (3.0, 7.0)	0.371
HbA1c, %	6.9 (5.5, 9.2)	6.9 (5.8, 8.8)	6.5 (5.4, 9.2)	7.1 (5.4, 9.2)	0.740
Fasting blood glucose, mmol/L	5.8 (4.8, 7.2)	5.2 (4.4, 6.4)	5.8 (4.8, 7.3)	6.1 (5.1, 7.9)	< 0.001
2-h postprandial blood glucose, mmol/L	9.7 (8.1, 12.7)	9.5 (8.0, 10.7)	9.7 (8.0, 12.7)	10.5 (8.5, 13.7)	0.023
Workday sleep duration, h	6.50 (6.00, 7.08)	6.50 (5.90, 7.10)	6.42 (6.00, 7.08)	6.58 (6.00, 7.08)	0.803
Free-day sleep duration, h	7.22 (6.67, 7.83)	7.20 (6.63, 7.96)	7.25 (6.63, 7.91)	7.23 (6.77, 7.64)	0.997
Weighted mean weekly sleep duration, h	6.70 (6.22, 7.25)	6.61 (6.24, 7.28)	6.68 (6.22, 7.36)	6.76 (6.21, 7.21)	0.908
Free-day–workday sleep-duration difference, h	0.72 (0.35, 1.03)	0.76 (0.37, 1.03)	0.75 (0.42, 1.00)	0.67 (0.30, 1.03)	0.710
PSQI score	7.0 (5.0, 9.0)	7.0 (4.0, 8.0)	8.0 (5.0, 10.0)	7.0 (6.0, 9.0)	0.030
PHQ-9 score	12.0 (8.0, 16.0)	10.0 (7.0, 13.3)	12.0 (7.0, 16.0)	14.0 (9.0, 17.5)	0.003
SDSCA score	35.0 (28.0, 44.0)	39.0 (31.0, 44.0)	35.0 (28.0, 44.0)	33.0 (26.0, 40.0)	0.004
Gender, *n* (%)					0.205
Male	189 (55.8)	45 (57.7)	82 (60.3)	62 (49.6)	
Female	150 (44.2)	33 (42.3)	54 (39.7)	63 (50.4)	

Continuous variables are presented as median (Q1, Q3), with quartiles calculated using the default SPSS FREQUENCIES method, and compared using the Kruskal–Wallis test; categorical variables are presented as *n* (%) and compared using Pearson's chi-square test. Percentages are column percentages. Further sociodemographic, clinical, and lifestyle characteristics and sleep-medication use are presented in Supplementary Table S1.

Social jetlag was < 1 h in 78 participants (23.0%), 1–2 h in 136 (40.1%), and >2 h in 125 (36.9%). Median PSQI, PHQ-9, and SDSCA scores were 7.0 (5.0, 9.0), 12.0 (8.0, 16.0), and 35.0 (28.0, 44.0), respectively. Age, FPG, 2-h PPG, PSQI, PHQ-9, and SDSCA differed across social jetlag groups (all *P* < 0.05). Workday sleep duration, free-day sleep duration, weighted mean weekly sleep duration, and the free-day–workday sleep-duration difference did not differ across groups (all *P* > 0.70; Table 1). Sleep-medication use frequency also did not differ across groups (*P* = 0.254; Supplementary Table S1).

### 3.2 Associations of social jetlag with sleep quality, depressive symptoms, and diabetes self-management

In separate adjusted regression models, greater social jetlag was associated with poorer sleep quality, more severe depressive symptoms, and lower diabetes self-management. For each additional h of social jetlag, the PSQI score was 1.352 points higher (*B* = 1.352, 95% CI 1.042 to 1.663, *P* < 0.001), and the PHQ-9 score was 3.772 points higher (*B* = 3.772, 95% CI 3.314 to 4.229, *P* < 0.001). The SDSCA score was 6.623 points lower (*B* = −6.623, 95% CI −7.566 to −5.679, *P* < 0.001).

### 3.3 Serial mediation analysis

The adjusted serial mediation model showed the estimated path coefficients (Figure 1; Table 2). Social jetlag was positively associated with PSQI score (*B* = 1.352, 95% CI 1.042 to 1.663, *P* < 0.001) and PHQ-9 score (*B* = 3.306, 95% CI 2.813 to 3.799, *P* < 0.001). PSQI score was positively associated with PHQ-9 score (*B* = 0.344, 95% CI 0.188 to 0.500, *P* < 0.001), whereas PHQ-9 score was negatively associated with SDSCA score (*B* = −0.675, 95% CI −0.892 to −0.457, *P* < 0.001). After PHQ-9 score was included in the model, PSQI score was not significantly associated with SDSCA score (*B* = −0.184, 95% CI −0.504 to 0.136, *P* = 0.260). The direct association of social jetlag with SDSCA score remained significant after PSQI and PHQ-9 scores were included in the model (*B* = −3.830, 95% CI −5.048 to −2.611, *P* < 0.001). Full regression coefficients for the primary serial mediation model are provided in Supplementary Table S2.

**Figure 1:**
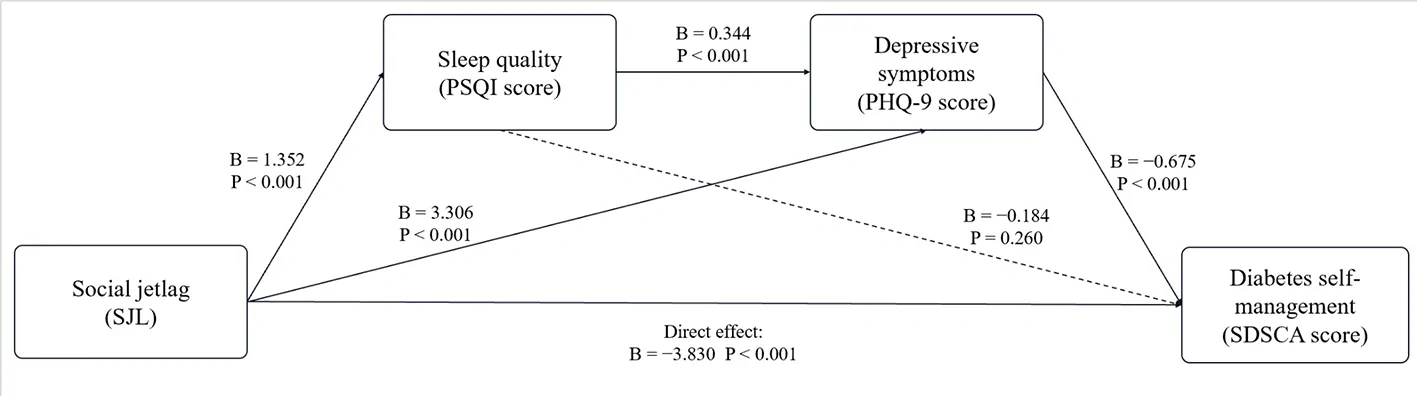
Adjusted serial mediation model linking social jetlag to diabetes self-management through sleep quality and depressive symptoms. Values are unstandardised regression coefficients (B) and two-sided *P* values. Solid arrows indicate statistically significant paths; the dashed arrow indicates the nonsignificant path from PSQI score to SDSCA score. All models were adjusted for age, gender, body mass index, duration of diabetes, self-reported number of diabetes-related complications, smoking frequency, drinking frequency, caffeine intake frequency, and napping-pattern indicator variables (no napping as the reference); the PHQ-9 model additionally included PSQI score, and the SDSCA model additionally included both PSQI and PHQ-9 scores. SJL, social jetlag; PSQI, Pittsburgh sleep quality index; PHQ-9, Patient Health Questionnaire-9; SDSCA, summary of diabetes self-care activities.

**Table 2:** Path coefficients from the adjusted serial mediation model.

Outcome	Predictor	*B*	SE	95% CI lower	95% CI upper	*P* value	Model note
PSQI score	Social jetlag (per 1-h increase)	1.352	0.158	1.042	1.663	< 0.001	*R*^2^ = 0.477
PHQ-9 score	Social jetlag (per 1-h increase)	3.306	0.251	2.813	3.799	< 0.001	*R*^2^ = 0.741
PHQ-9 score	PSQI score	0.344	0.079	0.188	0.500	< 0.001	
SDSCA score	Social jetlag (direct effect, per 1-h increase)	−3.830	0.619	−5.048	−2.611	< 0.001	*R*^2^ = 0.728
SDSCA score	PSQI score	−0.184	0.163	−0.504	0.136	0.260	
SDSCA score	PHQ-9 score	−0.675	0.111	−0.892	−0.457	< 0.001	

All models were adjusted for age, gender, body mass index, duration of diabetes, self-reported number of diabetes-related complications, smoking frequency, drinking frequency, caffeine intake frequency, and napping-pattern indicator variables (workdays only, free days only, and both workdays and free days; no napping as the reference). The PHQ-9 model additionally included PSQI score. The SDSCA model additionally included both PSQI and PHQ-9 scores. Full coefficients are provided in Supplementary Table S2.

The bootstrap analysis showed that the total indirect association between social jetlag and SDSCA score was significant (indirect effect = −2.793, BootSE = 0.486, bootstrap 95% CI −3.819 to −1.892; Table 3). The specific indirect association through depressive symptoms was significant (indirect effect = −2.230, BootSE = 0.400, bootstrap 95% CI −3.053 to −1.469). The serial indirect association through sleep quality and depressive symptoms was also significant (indirect effect = −0.314, BootSE = 0.099, bootstrap 95% CI −0.534 to −0.150). However, the specific indirect association through sleep quality was not significant (indirect effect = −0.249, BootSE = 0.214, bootstrap 95% CI −0.691 to 0.168).

**Table 3:** Total, direct, and indirect effects from the serial mediation model.

Effect	Unstandardised effect estimate	SE/BootSE	95% CI lower	95% CI upper	*P* value
Total effect: SJL → SDSCA	−6.623	0.480	−7.566	−5.679	< 0.001
Direct effect: SJL → SDSCA	−3.830	0.619	−5.048	−2.611	< 0.001
Total indirect effect	−2.793	0.486	−3.819	−1.892	
Indirect 1: SJL → PSQI → SDSCA	−0.249	0.214	−0.691	0.168	
Indirect 2: SJL → PHQ-9 → SDSCA	−2.230	0.400	−3.053	−1.469	
Serial indirect: SJL → PSQI → PHQ-9 → SDSCA	−0.314	0.099	−0.534	−0.150	

In the serial mediation model, PSQI was specified as the first mediator and PHQ-9 as the second mediator between social jetlag and SDSCA. Indirect effects were estimated using PROCESS Model 6 with 5,000 bootstrap resamples, 95% percentile bootstrap confidence intervals and a fixed random seed (12,345). Cases were sorted by participant ID and the SPSS MC random-number generator was used. Bootstrap confidence intervals that do not include zero indicate statistical significance. All models used the primary covariate set with napping entered as indicator variables. CI, confidence interval; PHQ-9, Patient Health Questionnaire-9; PSQI, Pittsburgh Sleep Quality Index; SDSCA, summary of diabetes self-care activities; SJL, social jetlag.

### 3.4 Sensitivity analyses

The serial indirect association remained statistically significant when education, marital status, and monthly per-capita income were added to the primary model (Supplementary Table S3), when HbA1c, fasting plasma glucose, and 2-h postprandial plasma glucose were additionally included in the S3 model (Supplementary Table S4), when the PHQ-9 sleep item was excluded (Supplementary Table S6), after adjustment for weighted mean weekly sleep duration and the free-day–workday sleep-duration difference (Supplementary Table S7), when the PSQI sleep-duration component was removed (Supplementary Table S8), when the PSQI sleep-medication component was removed (Supplementary Table S9), and after adjustment for recruitment site (Supplementary Table S10). The reverse-order serial indirect association was not statistically significant (Supplementary Table S5).

## 4 Discussion

The study examined the association between social jetlag and diabetes self-management in adults with T2D and the indirect associations involving sleep quality and depressive symptoms. Greater social jetlag was associated with poorer diabetes self-management. A significant specific indirect association was observed through depressive symptoms, while the indirect association through sleep quality alone was not significant. The serial indirect association involving sleep quality and depressive symptoms was significant. These findings describe statistical associations and do not establish temporal or causal pathways.

Our findings suggest that differences in sleep timing across the week may be relevant to diabetes self-management. The present finding extends previous evidence linking social jetlag to metabolic (11, 13) and psychological outcomes (35, 36). One possible explanation is that diabetes self-management behaviors must be performed repeatedly within regular daily schedules (37). These behaviors include dietary regulation, physical activity, medication adherence, and blood glucose monitoring (38). Differences in sleep timing between workdays and free days (10, 39) may make these schedules less stable (40, 41). This instability may make diabetes self-management behaviors more difficult to plan and maintain (9). In clinical practice, brief assessment of sleep timing across workdays and free days may help nurses identify routine-related barriers that are not captured by standard diabetes education (37, 42). Patients with substantial differences in sleep timing may benefit from self-management plans that are adapted to their weekly schedules.

We found that depressive symptoms may be an important psychological link between social jetlag and diabetes self-management. Previous studies have linked social jetlag to adverse psychological outcomes (35, 36). Previous studies also showed that depressive symptoms and emotional distress are associated with poorer diabetes self-management (20, 43) and lower treatment adherence (21). One possible explanation is that effective diabetes self-management requires sustained motivation, self-efficacy, planning, and problem-solving (3, 43). Depressive symptoms may reduce motivation and self-efficacy and impair planning and problem-solving (43, 44). Reduced motivation and self-efficacy, along with impaired planning and problem-solving, may make it harder for patients to maintain dietary regulation, physical activity, medication adherence, and blood glucose monitoring, even when they understand the recommended behaviors (2, 3). These findings support incorporating brief assessment of depressive symptoms into diabetes education and follow-up. Patients with elevated symptoms may require more structured self-management support or referral for further psychological assessment.

Our study showed that the specific indirect association through sleep quality alone was not significant. However, the serial indirect association involving sleep quality and depressive symptoms was significant. Poor sleep quality may disrupt daytime alertness, concentration, and daily routines, which are relevant to diabetes self-management (19, 42). One possible explanation for the different indirect associations is the overlap between poor sleep quality and depressive symptoms. These two constructs share manifestations such as fatigue, sleep disturbance, and impaired daytime functioning (16, 38). This overlap is also reflected in their measurement: the PSQI assesses daytime dysfunction, whereas the PHQ-9 includes sleep disturbance and fatigue (28, 29). Depressive symptoms may more directly reflect difficulties in initiating and sustaining self-management behaviors. Therefore, after depressive symptoms were included in the model, sleep quality provided limited additional explanatory information about diabetes self-management. The close relationship between poor sleep quality and depressive symptoms may nevertheless explain why sleep quality remained part of the serial statistical association. In practice, sleep quality should not be assessed in isolation. Joint assessment of sleep quality and depressive symptoms may provide a clearer understanding of the sleep-related and emotional difficulties associated with poor self-management.

This study has several strengths. Social jetlag was calculated from both workday and free-day sleep midpoints, allowing sleep-timing misalignment to be quantified directly. The serial mediation model distinguished the direct association from specific and serial indirect associations. In addition, the main findings remained consistent across broader covariate adjustment, sleep-duration analyses, and modified mediator scores; the recruitment-site-adjusted model yielded a similar serial estimate.

The study has four limitations. First, the cross-sectional design does not establish temporal order or causality. Second, the study included only employed adults aged 18–60 years recruited by convenience sampling from tertiary public general hospitals in a single region of China. Therefore, the generalisability of these findings may be limited beyond the employed adult population and hospital settings represented in this study. Third, reliance on questionnaire-based measures may have introduced recall or reporting bias, particularly because participants self-reported the number of previously diagnosed diabetes-related complications. In addition, the absence of data on specific complication types and independent diagnostic verification may have introduced measurement error or misclassification. Glycaemic values were entered with reference to medical records or laboratory reports from the preceding 3 months, but exact test dates and assay methods were not recorded. Fourth, data on diabetes treatment regimens and complexity, detailed comorbidities, diagnosed sleep disorders, psychiatric history, chronic pain, and specific psychotropic medication use were not collected. Consequently, residual confounding remains possible. Although the PSQI captured the frequency of medication use to aid sleep, specific medication classes were unavailable.

## 5 Conclusion

Among adults with T2D, greater social jetlag was associated with poorer diabetes self-management. Statistical indirect associations were also observed through depressive symptoms alone and through the serial pathway involving sleep quality and depressive symptoms. Because the data were cross-sectional, these associations cannot establish temporal ordering or causal mediation. Nurses should therefore consider sleep timing, sleep quality, and depressive symptoms together when assessing potential barriers to diabetes self-management. Longitudinal studies are needed to clarify the temporal relationships among these variables.

## Data Availability

The raw data supporting the conclusions of this article will be made available by the authors, without undue reservation.
