# Supplementary material for: Association between social jetlag and diabetes self-management in adults with type 2 diabetes: a cross-sectional study with serial mediation analysis
Source: Front Public Health. 2026 Oct 16;14:1979499. doi: 10.3389/fpubh.2026.1979499 (PMC13719569; doi:10.3389/fpubh.2026.1979499)
Supplement: Supplementary file 1 [file Table_1.docx]

**Supplementary material**

Supplementary Table S1 presents sociodemographic, clinical, and lifestyle characteristics and sleep-medication use by social jetlag group. Supplementary Table S2 presents full coefficients for the primary serial mediation model, with napping entered as a nominal variable using indicator variables. Supplementary Tables S3–S10 present the sensitivity analyses. All analyses were performed using IBM SPSS Statistics version 27.0.1 and PROCESS version 5.0 (Model 6). All coefficients are unstandardised. The 95% percentile bootstrap confidence intervals for indirect effects were estimated using 5,000 resamples with a fixed random seed (12345), with cases sorted by participant ID and the SPSS MC random-number generator used throughout.

**Supplementary Table S1. Sociodemographic, clinical, and lifestyle characteristics and sleep-medication use according to social jetlag group**

Categorical variables are presented as n (%) and compared using Pearson's chi-square test. Percentages are column percentages.

| **Characteristic** | **Overall (N = 339)** | **<1 h (n = 78)** | **1–2 h (n = 136)** | **>2 h (n = 125)** | **P value** |
| --- | --- | --- | --- | --- | --- |
| Age group, n (%) |  |  |  |  | <0.001 |
| 18–29 years | 49 (14.5) | 2 (2.6) | 19 (14.0) | 28 (22.4) |  |
| 30–39 years | 132 (38.9) | 24 (30.8) | 53 (39.0) | 55 (44.0) |  |
| 40–49 years | 119 (35.1) | 36 (46.2) | 49 (36.0) | 34 (27.2) |  |
| 50–60 years | 39 (11.5) | 16 (20.5) | 15 (11.0) | 8 (6.4) |  |
| Education, n (%) |  |  |  |  | 0.025 |
| Primary school or below | 48 (14.2) | 12 (15.4) | 20 (14.7) | 16 (12.8) |  |
| Junior high school | 80 (23.6) | 20 (25.6) | 40 (29.4) | 20 (16.0) |  |
| Senior high/technical school | 77 (22.7) | 25 (32.1) | 26 (19.1) | 26 (20.8) |  |
| Junior college | 97 (28.6) | 17 (21.8) | 34 (25.0) | 46 (36.8) |  |
| Bachelor degree or above | 37 (10.9) | 4 (5.1) | 16 (11.8) | 17 (13.6) |  |
| Marital status, n (%) |  |  |  |  | 0.013 |
| Unmarried | 95 (28.0) | 11 (14.1) | 35 (25.7) | 49 (39.2) |  |
| Married | 206 (60.8) | 57 (73.1) | 85 (62.5) | 64 (51.2) |  |
| Divorced | 29 (8.6) | 7 (9.0) | 13 (9.6) | 9 (7.2) |  |
| Widowed | 9 (2.7) | 3 (3.8) | 3 (2.2) | 3 (2.4) |  |
| Monthly per capita income (RMB), n (%) |  |  |  |  | 0.023 |
| ≤1000 | 16 (4.7) | 6 (7.7) | 5 (3.7) | 5 (4.0) |  |
| 1001–3000 | 74 (21.8) | 26 (33.3) | 31 (22.8) | 17 (13.6) |  |
| 3001–5000 | 124 (36.6) | 27 (34.6) | 51 (37.5) | 46 (36.8) |  |
| 5001–7000 | 87 (25.7) | 14 (17.9) | 36 (26.5) | 37 (29.6) |  |
| >7000 | 38 (11.2) | 5 (6.4) | 13 (9.6) | 20 (16.0) |  |
| Diabetes-related complications, n (%) |  |  |  |  | 0.120 |
| 0 | 191 (56.3) | 50 (64.1) | 72 (52.9) | 69 (55.2) |  |
| 1 | 67 (19.8) | 14 (17.9) | 27 (19.9) | 26 (20.8) |  |
| 2 | 63 (18.6) | 10 (12.8) | 25 (18.4) | 28 (22.4) |  |
| ≥3 | 18 (5.3) | 4 (5.1) | 12 (8.8) | 2 (1.6) |  |
| Smoking frequency in past year, n (%) |  |  |  |  | 0.107 |
| None | 108 (31.9) | 31 (39.7) | 41 (30.1) | 36 (28.8) |  |
| <1 time/week | 38 (11.2) | 6 (7.7) | 11 (8.1) | 21 (16.8) |  |
| 1–3 times/week | 88 (26.0) | 18 (23.1) | 33 (24.3) | 37 (29.6) |  |
| 4–6 times/week | 78 (23.0) | 17 (21.8) | 40 (29.4) | 21 (16.8) |  |
| ≥7 times/week | 27 (8.0) | 6 (7.7) | 11 (8.1) | 10 (8.0) |  |
| Drinking frequency in past year, n (%) |  |  |  |  | 0.544 |
| None | 74 (21.8) | 20 (25.6) | 25 (18.4) | 29 (23.2) |  |
| <1 time/week | 100 (29.5) | 23 (29.5) | 39 (28.7) | 38 (30.4) |  |
| 1–3 times/week | 69 (20.4) | 13 (16.7) | 34 (25.0) | 22 (17.6) |  |
| 4–6 times/week | 59 (17.4) | 17 (21.8) | 22 (16.2) | 20 (16.0) |  |
| ≥7 times/week | 37 (10.9) | 5 (6.4) | 16 (11.8) | 16 (12.8) |  |
| Caffeine intake frequency in past year, n (%) |  |  |  |  | 0.199 |
| None | 36 (10.6) | 11 (14.1) | 16 (11.8) | 9 (7.2) |  |
| <1 time/week | 74 (21.8) | 17 (21.8) | 31 (22.8) | 26 (20.8) |  |
| 1–3 times/week | 98 (28.9) | 30 (38.5) | 34 (25.0) | 34 (27.2) |  |
| 4–6 times/week | 85 (25.1) | 14 (17.9) | 34 (25.0) | 37 (29.6) |  |
| ≥7 times/week | 46 (13.6) | 6 (7.7) | 21 (15.4) | 19 (15.2) |  |
| Napping habit in past year, n (%) |  |  |  |  | 0.657 |
| None | 126 (37.2) | 23 (29.5) | 50 (36.8) | 53 (42.4) |  |
| Workdays only | 108 (31.9) | 28 (35.9) | 43 (31.6) | 37 (29.6) |  |
| Free days only | 29 (8.6) | 6 (7.7) | 12 (8.8) | 11 (8.8) |  |
| Both workdays and free days | 76 (22.4) | 21 (26.9) | 31 (22.8) | 24 (19.2) |  |
| Sleep-medication use in previous month, n (%) |  |  |  |  | 0.254 |
| None | 61 (18.0) | 14 (17.9) | 24 (17.6) | 23 (18.4) |  |
| <1 time/week | 213 (62.8) | 56 (71.8) | 79 (58.1) | 78 (62.4) |  |
| 1–2 times/week | 61 (18.0) | 7 (9.0) | 32 (23.5) | 22 (17.6) |  |
| ≥3 times/week | 4 (1.2) | 1 (1.3) | 1 (0.7) | 2 (1.6) |  |

Abbreviations: SJL, social jetlag.

**Supplementary Table S2. Full regression coefficients for the primary serial mediation model**

Panel A presents the PSQI outcome model (R² = 0.477; adjusted R² = 0.458), Panel B the PHQ-9 outcome model (R² = 0.741; adjusted R² = 0.730), and Panel C the SDSCA outcome model (R² = 0.728; adjusted R² = 0.717). Primary covariates were age, gender, body mass index, duration of diabetes, self-reported number of diabetes-related complications, smoking frequency, drinking frequency, caffeine intake frequency, and napping pattern. Smoking, drinking, and caffeine intake were entered as ordered frequency variables; napping was entered as three indicator variables with no napping as the reference category. The PHQ-9 model additionally included PSQI score; the SDSCA model additionally included both PSQI and PHQ-9 scores. Unstandardised coefficients are shown.

| **Predictor** | **B** | **SE** | **95% CI lower** | **95% CI upper** | **P value** | **VIF** |
| --- | --- | --- | --- | --- | --- | --- |
| Panel A. Outcome: PSQI score | | | | | | |
| Constant | −9.186 | 1.173 | −11.494 | −6.878 | <0.001 |  |
| SJL | 1.352 | 0.158 | 1.042 | 1.663 | <0.001 | 1.100 |
| Age | 0.196 | 0.014 | 0.169 | 0.223 | <0.001 | 1.088 |
| Gender | −0.162 | 0.231 | −0.617 | 0.293 | 0.485 | 1.028 |
| Body mass index | 0.230 | 0.035 | 0.162 | 0.298 | <0.001 | 1.024 |
| Duration of diabetes | 0.114 | 0.032 | 0.051 | 0.177 | <0.001 | 1.036 |
| Number of complications | 0.105 | 0.122 | −0.135 | 0.345 | 0.390 | 1.029 |
| Smoking | 0.003 | 0.086 | −0.166 | 0.172 | 0.968 | 1.038 |
| Drinking | 0.101 | 0.089 | −0.073 | 0.275 | 0.256 | 1.016 |
| Caffeine intake | 0.096 | 0.096 | −0.092 | 0.285 | 0.315 | 1.021 |
| Napping: workdays only vs none | 0.059 | 0.275 | −0.482 | 0.601 | 0.830 | 1.282 |
| Napping: free days only vs none | 0.182 | 0.431 | −0.666 | 1.029 | 0.673 | 1.132 |
| Napping: both vs none | 0.488 | 0.306 | −0.115 | 1.091 | 0.112 | 1.272 |
| Panel B. Outcome: PHQ-9 score | | | | | | |
| Constant | −23.397 | 1.833 | −27.002 | −19.791 | <0.001 |  |
| SJL | 3.306 | 0.251 | 2.813 | 3.799 | <0.001 | 1.347 |
| PSQI score | 0.344 | 0.079 | 0.188 | 0.500 | <0.001 | 1.914 |
| Age | 0.439 | 0.025 | 0.390 | 0.488 | <0.001 | 1.766 |
| Gender | 0.684 | 0.332 | 0.032 | 1.337 | 0.040 | 1.029 |
| Body mass index | 0.370 | 0.053 | 0.266 | 0.474 | <0.001 | 1.162 |
| Duration of diabetes | 0.063 | 0.047 | −0.029 | 0.156 | 0.176 | 1.077 |
| Number of complications | 0.294 | 0.175 | −0.050 | 0.639 | 0.093 | 1.031 |
| Smoking | −0.064 | 0.123 | −0.306 | 0.178 | 0.604 | 1.038 |
| Drinking | 0.321 | 0.127 | 0.071 | 0.571 | 0.012 | 1.020 |
| Caffeine intake | −0.262 | 0.137 | −0.533 | 0.008 | 0.057 | 1.024 |
| Napping: workdays only vs none | 0.980 | 0.395 | 0.204 | 1.756 | 0.014 | 1.283 |
| Napping: free days only vs none | −0.564 | 0.618 | −1.780 | 0.651 | 0.362 | 1.133 |
| Napping: both vs none | −0.146 | 0.441 | −1.013 | 0.721 | 0.741 | 1.282 |
| Panel C. Outcome: SDSCA score | | | | | | |
| Constant | 91.390 | 4.480 | 82.578 | 100.203 | <0.001 |  |
| SJL | −3.830 | 0.619 | −5.048 | −2.611 | <0.001 | 2.069 |
| PSQI score | −0.184 | 0.163 | −0.504 | 0.136 | 0.260 | 2.025 |
| PHQ-9 score | −0.675 | 0.111 | −0.892 | −0.457 | <0.001 | 3.855 |
| Age | −0.610 | 0.070 | −0.747 | −0.473 | <0.001 | 3.436 |
| Gender | −1.000 | 0.666 | −2.310 | 0.310 | 0.134 | 1.043 |
| Body mass index | −0.589 | 0.113 | −0.812 | −0.367 | <0.001 | 1.337 |
| Duration of diabetes | −0.023 | 0.094 | −0.207 | 0.162 | 0.809 | 1.083 |
| Number of complications | −0.309 | 0.351 | −0.998 | 0.381 | 0.379 | 1.040 |
| Smoking | −0.007 | 0.246 | −0.491 | 0.476 | 0.976 | 1.039 |
| Drinking | 0.184 | 0.256 | −0.319 | 0.688 | 0.472 | 1.040 |
| Caffeine intake | −0.563 | 0.276 | −1.105 | −0.020 | 0.042 | 1.036 |
| Napping: workdays only vs none | 0.000 | 0.795 | −1.563 | 1.563 | 1.000 | 1.307 |
| Napping: free days only vs none | 2.273 | 1.234 | −0.154 | 4.700 | 0.066 | 1.136 |
| Napping: both vs none | 0.031 | 0.879 | −1.699 | 1.761 | 0.972 | 1.282 |

Note. CI, confidence interval; PHQ-9, Patient Health Questionnaire-9; PSQI, Pittsburgh Sleep Quality Index; SDSCA, Summary of Diabetes Self-Care Activities; SJL, social jetlag.

**Sensitivity analyses for the serial mediation model**

The primary serial mediation model specified PSQI as the first mediator and PHQ-9 as the second mediator between social jetlag and SDSCA. Supplementary Tables S3 and S4 present analyses using broader covariate adjustment sets. Supplementary Table S5 reverses the mediator order, Supplementary Table S6 excludes the PHQ-9 sleep item, Supplementary Table S7 additionally adjusts for sleep-duration measures, Supplementary Table S8 excludes the PSQI sleep-duration component, Supplementary Table S9 excludes the PSQI sleep-medication component, and Supplementary Table S10 additionally adjusts for recruitment site. All coefficients are unstandardised. The 95% percentile bootstrap confidence intervals for indirect effects were estimated using 5,000 bootstrap resamples with a fixed random seed (12345); the participant-ID order and SPSS MC random-number generator specified above were retained in every analysis.

**Supplementary Table S3. Sensitivity analysis additionally adjusting for sociodemographic variables**

Sensitivity analysis 1 additionally adjusted for education, monthly per capita income, and marital-status dummy variables (unmarried, divorced, and widowed; married as the reference group), in addition to the primary covariate set.

| **Effect / model component** | **Estimate** | **SE / BootSE** | **95% CI lower** | **95% CI upper** | **P value** | **Model note** |
| --- | --- | --- | --- | --- | --- | --- |
| PSQI score: SJL (per 1-h increase) | 1.476 | 0.164 | 1.154 | 1.799 | <0.001 |  |
| PHQ-9 score: SJL (per 1-h increase) | 3.345 | 0.267 | 2.819 | 3.870 | <0.001 |  |
| PHQ-9 score: PSQI score | 0.345 | 0.081 | 0.185 | 0.505 | <0.001 |  |
| SDSCA score: SJL direct effect (per 1-h increase) | −3.891 | 0.652 | −5.174 | −2.609 | <0.001 |  |
| SDSCA score: PSQI score | −0.178 | 0.167 | −0.507 | 0.150 | 0.286 |  |
| SDSCA score: PHQ-9 score | −0.673 | 0.112 | −0.893 | −0.453 | <0.001 |  |
| Total effect: SJL → SDSCA | −6.747 | 0.506 | −7.743 | −5.751 | <0.001 |  |
| Direct effect: SJL → SDSCA | −3.891 | 0.652 | −5.174 | −2.609 | <0.001 |  |
| Total indirect effect | −2.855 | 0.500 | −3.905 | −1.929 |  | Bootstrap CI |
| Indirect effect 1: SJL → PSQI score → SDSCA | −0.263 | 0.232 | −0.728 | 0.189 |  | Bootstrap CI |
| Indirect effect 2: SJL → PHQ-9 score → SDSCA | −2.250 | 0.406 | −3.101 | −1.486 |  | Bootstrap CI |
| Serial indirect effect: SJL → PSQI score → PHQ-9 score → SDSCA | −0.342 | 0.110 | −0.584 | −0.160 |  | Bootstrap CI |

Note. Primary covariates were age, gender, body mass index, duration of diabetes, self-reported number of diabetes-related complications, smoking frequency, drinking frequency, caffeine intake frequency, and napping-pattern indicator variables (no napping as the reference). The PHQ-9 model additionally included PSQI score; the SDSCA model additionally included both PSQI and PHQ-9 scores. CI, confidence interval; PHQ-9, Patient Health Questionnaire-9; PSQI, Pittsburgh Sleep Quality Index; SDSCA, Summary of Diabetes Self-Care Activities; SJL, social jetlag.

Supplementary Table S4. Sensitivity analysis additionally adjusting for sociodemographic variables and glycaemic indicators

Sensitivity analysis 2 additionally adjusted for education, monthly per capita income, marital-status dummy variables, HbA1c, fasting plasma glucose, and 2-h postprandial plasma glucose, in addition to the primary covariate set. Glycaemic indicators were treated as a supplementary robustness adjustment rather than established confounders because they may partly lie downstream of sleep timing and self-management behaviours.

| **Effect / model component** | **Estimate** | **SE / BootSE** | **95% CI lower** | **95% CI upper** | **P value** | **Model note** |
| --- | --- | --- | --- | --- | --- | --- |
| PSQI score: SJL (per 1-h increase) | 1.425 | 0.170 | 1.089 | 1.760 | <0.001 |  |
| PHQ-9 score: SJL (per 1-h increase) | 3.324 | 0.275 | 2.784 | 3.865 | <0.001 |  |
| PHQ-9 score: PSQI score | 0.341 | 0.082 | 0.180 | 0.502 | <0.001 |  |
| SDSCA score: SJL direct effect (per 1-h increase) | −3.684 | 0.660 | −4.983 | −2.386 | <0.001 |  |
| SDSCA score: PSQI score | −0.151 | 0.167 | −0.479 | 0.178 | 0.367 |  |
| SDSCA score: PHQ-9 score | −0.676 | 0.112 | −0.895 | −0.456 | <0.001 |  |
| Total effect: SJL → SDSCA | −6.474 | 0.524 | −7.505 | −5.442 | <0.001 |  |
| Direct effect: SJL → SDSCA | −3.684 | 0.660 | −4.983 | −2.386 | <0.001 |  |
| Total indirect effect | −2.789 | 0.496 | −3.842 | −1.872 |  | Bootstrap CI |
| Indirect effect 1: SJL → PSQI score → SDSCA | −0.215 | 0.222 | −0.666 | 0.222 |  | Bootstrap CI |
| Indirect effect 2: SJL → PHQ-9 score → SDSCA | −2.247 | 0.414 | −3.109 | −1.466 |  | Bootstrap CI |
| Serial indirect effect: SJL → PSQI score → PHQ-9 score → SDSCA | −0.328 | 0.109 | −0.566 | −0.148 |  | Bootstrap CI |

Note. Primary covariates were age, gender, body mass index, duration of diabetes, self-reported number of diabetes-related complications, smoking frequency, drinking frequency, caffeine intake frequency, and napping-pattern indicator variables (no napping as the reference). The PHQ-9 model additionally included PSQI score; the SDSCA model additionally included both PSQI and PHQ-9 scores. CI, confidence interval; HbA1c, glycated haemoglobin; PHQ-9, Patient Health Questionnaire-9; PSQI, Pittsburgh Sleep Quality Index; SDSCA, Summary of Diabetes Self-Care Activities; SJL, social jetlag.

**Supplementary Table S5. Sensitivity analysis reversing the order of the two mediators**

The mediator order was reversed, with PHQ-9 entered before PSQI. The primary covariate set, including napping-pattern indicator variables, was unchanged. Indirect effects were estimated using 5,000 bootstrap resamples with a fixed random seed (12345).

| **Effect** | **Estimate** | **SE / BootSE** | **95% CI lower** | **95% CI upper** | **P value** |
| --- | --- | --- | --- | --- | --- |
| Total effect: SJL → SDSCA | −6.623 | 0.480 | −7.566 | −5.679 | <0.001 |
| Direct effect: SJL → SDSCA | −3.830 | 0.619 | −5.048 | −2.611 | <0.001 |
| Total indirect effect | −2.793 | 0.486 | −3.819 | −1.892 |  |
| Indirect 1: SJL → PHQ-9 → SDSCA | −2.544 | 0.450 | −3.475 | −1.683 |  |
| Indirect 2: SJL → PSQI → SDSCA | −0.138 | 0.128 | −0.424 | 0.093 |  |
| Serial indirect: SJL → PHQ-9 → PSQI → SDSCA | −0.110 | 0.100 | −0.330 | 0.074 |  |

Note. Bootstrap confidence intervals that do not include zero indicate statistical significance. CI, confidence interval; PHQ-9, Patient Health Questionnaire-9; PSQI, Pittsburgh Sleep Quality Index; SDSCA, Summary of Diabetes Self-Care Activities; SJL, social jetlag.

**Supplementary Table S6. Sensitivity analysis excluding the PHQ-9 sleep item**

The primary serial model was repeated using an exploratory depressive-symptom score calculated as the PHQ-9 total score minus the sleep-disturbance item (item 3; possible range 0–24). The hypothesised mediator order, with PSQI entered before the depressive-symptom score, was retained. The primary covariate set, including napping-pattern indicator variables, was unchanged. Indirect effects were estimated using 5,000 bootstrap resamples with a fixed random seed (12345).

| **Effect** | **Estimate** | **SE / BootSE** | **95% CI lower** | **95% CI upper** | **P value** |
| --- | --- | --- | --- | --- | --- |
| Total effect: SJL → SDSCA | −6.623 | 0.480 | −7.566 | −5.679 | <0.001 |
| Direct effect: SJL → SDSCA | −4.116 | 0.621 | −5.337 | −2.895 | <0.001 |
| Total indirect effect | −2.507 | 0.471 | −3.498 | −1.625 |  |
| Indirect 1: SJL → PSQI → SDSCA | −0.278 | 0.220 | −0.727 | 0.148 |  |
| Indirect 2: SJL → depressive symptoms excluding the PHQ-9 sleep item → SDSCA | −1.944 | 0.384 | −2.725 | −1.219 |  |
| Serial indirect: SJL → PSQI → depressive symptoms excluding the PHQ-9 sleep item → SDSCA | −0.285 | 0.094 | −0.496 | −0.132 |  |

Note. The depressive-symptom score excluding the PHQ-9 sleep item was used only as an exploratory sensitivity measure and was not treated as a separate validated scale. Bootstrap confidence intervals that do not include zero indicate statistical significance. CI, confidence interval; PHQ-9, Patient Health Questionnaire-9; PSQI, Pittsburgh Sleep Quality Index; SDSCA, Summary of Diabetes Self-Care Activities; SJL, social jetlag.

**Supplementary Table S7. Sensitivity analysis additionally adjusting for sleep-duration measures**

The primary serial model was repeated with additional adjustment for weighted mean weekly sleep duration and the free-day–workday sleep-duration difference. The primary covariate set was otherwise unchanged.

| **Effect / model component** | **Estimate** | **SE / BootSE** | **95% CI lower** | **95% CI upper** | **P value** | **Model note** |
| --- | --- | --- | --- | --- | --- | --- |
| PSQI score: SJL (per 1-h increase) | 1.348 | 0.158 | 1.038 | 1.658 | <0.001 |  |
| PHQ-9 score: SJL (per 1-h increase) | 3.312 | 0.252 | 2.817 | 3.807 | <0.001 |  |
| PHQ-9 score: PSQI score | 0.339 | 0.080 | 0.181 | 0.497 | <0.001 |  |
| SDSCA score: SJL direct effect (per 1-h increase) | −3.807 | 0.621 | −5.029 | −2.585 | <0.001 |  |
| SDSCA score: PSQI score | −0.181 | 0.164 | −0.503 | 0.142 | 0.271 |  |
| SDSCA score: PHQ-9 score | −0.674 | 0.111 | −0.892 | −0.456 | <0.001 |  |
| Total effect: SJL → SDSCA | −6.592 | 0.481 | −7.538 | −5.646 | <0.001 |  |
| Direct effect: SJL → SDSCA | −3.807 | 0.621 | −5.029 | −2.585 | <0.001 |  |
| Total indirect effect | −2.786 | 0.476 | −3.769 | −1.895 |  | Bootstrap CI |
| Indirect effect 1: SJL → PSQI score → SDSCA | −0.244 | 0.217 | −0.676 | 0.182 |  | Bootstrap CI |
| Indirect effect 2: SJL → PHQ-9 score → SDSCA | −2.234 | 0.397 | −3.050 | −1.461 |  | Bootstrap CI |
| Serial indirect effect: SJL → PSQI score → PHQ-9 score → SDSCA | −0.308 | 0.096 | −0.518 | −0.149 |  | Bootstrap CI |

Note. Weighted mean weekly sleep duration = [(workday sleep duration × number of workdays) + (free-day sleep duration × number of free days)]/7. CI, confidence interval; PHQ-9, Patient Health Questionnaire-9; PSQI, Pittsburgh Sleep Quality Index; SDSCA, Summary of Diabetes Self-Care Activities; SJL, social jetlag.

**Supplementary Table S8. Sensitivity analysis excluding the PSQI sleep-duration component**

The primary serial model was repeated using a modified PSQI score calculated by subtracting the sleep-duration component from the original PSQI total score. The hypothesised mediator order and primary covariate set were unchanged.

| **Effect / model component** | **Estimate** | **SE / BootSE** | **95% CI lower** | **95% CI upper** | **P value** | **Model note** |
| --- | --- | --- | --- | --- | --- | --- |
| Modified PSQI score: SJL (per 1-h increase) | 1.203 | 0.142 | 0.924 | 1.482 | <0.001 |  |
| PHQ-9 score: SJL (per 1-h increase) | 3.347 | 0.251 | 2.853 | 3.842 | <0.001 |  |
| PHQ-9 score: Modified PSQI score | 0.353 | 0.089 | 0.178 | 0.527 | <0.001 |  |
| SDSCA score: SJL direct effect (per 1-h increase) | −3.842 | 0.621 | −5.063 | −2.621 | <0.001 |  |
| SDSCA score: Modified PSQI score | −0.178 | 0.181 | −0.534 | 0.177 | 0.325 |  |
| SDSCA score: PHQ-9 score | −0.680 | 0.110 | −0.897 | −0.464 | <0.001 |  |
| Total effect: SJL → SDSCA | −6.623 | 0.480 | −7.566 | −5.679 | <0.001 |  |
| Direct effect: SJL → SDSCA | −3.842 | 0.621 | −5.063 | −2.621 | <0.001 |  |
| Total indirect effect | −2.781 | 0.487 | −3.803 | −1.888 |  | Bootstrap CI |
| Indirect effect 1: SJL → Modified PSQI score → SDSCA | −0.214 | 0.217 | −0.655 | 0.210 |  | Bootstrap CI |
| Indirect effect 2: SJL → PHQ-9 score → SDSCA | −2.278 | 0.412 | −3.140 | −1.501 |  | Bootstrap CI |
| Serial indirect effect: SJL → Modified PSQI score → PHQ-9 score → SDSCA | −0.289 | 0.095 | −0.499 | −0.132 |  | Bootstrap CI |

Note. The modified PSQI score was used only for sensitivity analysis and was not treated as a separate validated scale. CI, confidence interval; PHQ-9, Patient Health Questionnaire-9; PSQI, Pittsburgh Sleep Quality Index; SDSCA, Summary of Diabetes Self-Care Activities; SJL, social jetlag.

**Supplementary Table S9. Sensitivity analysis excluding the PSQI sleep-medication component**

The primary serial model was repeated using a modified PSQI score calculated by subtracting the sleep-medication component from the original PSQI total score. The hypothesised mediator order and primary covariate set were unchanged.

| **Effect / model component** | **Estimate** | **SE / BootSE** | **95% CI lower** | **95% CI upper** | **P value** | **Model note** |
| --- | --- | --- | --- | --- | --- | --- |
| Modified PSQI score: SJL (per 1-h increase) | 1.196 | 0.139 | 0.923 | 1.470 | <0.001 |  |
| PHQ-9 score: SJL (per 1-h increase) | 3.370 | 0.253 | 2.872 | 3.867 | <0.001 |  |
| PHQ-9 score: Modified PSQI score | 0.336 | 0.091 | 0.157 | 0.515 | <0.001 |  |
| SDSCA score: SJL direct effect (per 1-h increase) | −3.788 | 0.622 | −5.012 | −2.565 | <0.001 |  |
| SDSCA score: Modified PSQI score | −0.242 | 0.184 | −0.603 | 0.119 | 0.188 |  |
| SDSCA score: PHQ-9 score | −0.675 | 0.110 | −0.891 | −0.459 | <0.001 |  |
| Total effect: SJL → SDSCA | −6.623 | 0.480 | −7.566 | −5.679 | <0.001 |  |
| Direct effect: SJL → SDSCA | −3.788 | 0.622 | −5.012 | −2.565 | <0.001 |  |
| Total indirect effect | −2.834 | 0.491 | −3.876 | −1.917 |  | Bootstrap CI |
| Indirect effect 1: SJL → Modified PSQI score → SDSCA | −0.290 | 0.212 | −0.724 | 0.109 |  | Bootstrap CI |
| Indirect effect 2: SJL → PHQ-9 score → SDSCA | −2.274 | 0.407 | −3.115 | −1.492 |  | Bootstrap CI |
| Serial indirect effect: SJL → Modified PSQI score → PHQ-9 score → SDSCA | −0.271 | 0.095 | −0.480 | −0.118 |  | Bootstrap CI |

Note. The modified PSQI score was used only for sensitivity analysis and was not treated as a separate validated scale. CI, confidence interval; PHQ-9, Patient Health Questionnaire-9; PSQI, Pittsburgh Sleep Quality Index; SDSCA, Summary of Diabetes Self-Care Activities; SJL, social jetlag.

Supplementary Table S10. Sensitivity analysis additionally adjusting for recruitment site

The primary serial model was repeated with recruitment site represented by indicator variables. The General Hospital of Ningxia Medical University was the reference category, and all primary covariates were retained.

Note. Recruitment totals: General Hospital of Ningxia Medical University (n = 127), Wuzhong People’s Hospital (n = 111), and People’s Hospital of Ningxia Hui Autonomous Region (n = 101). CI, confidence interval; PHQ-9, Patient Health Questionnaire-9; PSQI, Pittsburgh Sleep Quality Index; SDSCA, Summary of Diabetes Self-Care Activities; SJL, social jetlag.

| **Effect / model component** | **Estimate** | **SE / BootSE** | **95% CI lower** | **95% CI upper** | **P value** | **Model note** |
| --- | --- | --- | --- | --- | --- | --- |
| PSQI score: SJL (per 1-h increase) | 1.360 | 0.160 | 1.046 | 1.674 | <0.001 |  |
| PHQ-9 score: SJL (per 1-h increase) | 3.299 | 0.253 | 2.802 | 3.796 | <0.001 |  |
| PHQ-9 score: PSQI score | 0.347 | 0.079 | 0.191 | 0.504 | <0.001 |  |
| SDSCA score: SJL direct effect (per 1-h increase) | −3.733 | 0.622 | −4.956 | −2.510 | <0.001 |  |
| SDSCA score: PSQI score | −0.178 | 0.163 | −0.499 | 0.142 | 0.274 |  |
| SDSCA score: PHQ-9 score | −0.684 | 0.111 | −0.902 | −0.467 | <0.001 |  |
| Total effect: SJL → SDSCA | −6.557 | 0.484 | −7.509 | −5.605 | <0.001 |  |
| Direct effect: SJL → SDSCA | −3.733 | 0.622 | −4.956 | −2.510 | <0.001 |  |
| Total indirect effect | −2.824 | 0.486 | −3.848 | −1.933 |  | Bootstrap CI |
| Indirect effect 1: SJL → PSQI score → SDSCA | −0.242 | 0.216 | −0.687 | 0.180 |  | Bootstrap CI |
| Indirect effect 2: SJL → PHQ-9 score → SDSCA | −2.258 | 0.398 | −3.069 | −1.499 |  | Bootstrap CI |
| Serial indirect effect: SJL → PSQI score → PHQ-9 score → SDSCA | −0.323 | 0.103 | −0.556 | −0.155 |  | Bootstrap CI |
